# Differential immunoglobulin and complement levels in leprosy prior to development of reversal reaction and erythema nodosum leprosum

**DOI:** 10.1371/journal.pntd.0007089

**Published:** 2019-01-28

**Authors:** Francianne M. Amorim, Maurício L. Nobre, Larissa S. Nascimento, Alesson M. Miranda, Glória R. G. Monteiro, Francisco P. Freire-Neto, Maria do Carmo Palmeira Queiroz, José W. Queiroz, Malcolm S. Duthie, Marcos R. Costa, Steven G. Reed, Warren D. Johnson, Kathryn M. Dupnik, Selma M. B. Jeronimo

**Affiliations:** 1 Department of Biochemistry, Biosciences Center, Federal University of Rio Grande do Norte, Natal, Rio Grande do Norte, Brazil; 2 Institute of Tropical Medicine of Rio Grande do Norte, Federal University of Rio Grande do Norte, Natal, Rio Grande do Norte, Brazil; 3 Giselda Trigueiro Hospital, State Health Secretariat, Rio Grande do Norte; Natal, Rio Grande do Norte, Brazil; 4 Department of Internal Medicine, Health Science Center and Onofre Lopes University Hospital, Federal University of Rio Grande do Norte, Natal, Rio Grande do Norte, Brazil; 5 Infectious Disease Research Institute, Seattle, Washington, United States of America; 6 Brain Institute, Federal University of Rio Grande do Norte, Natal, Rio Grande do Norte, Brazil; 7 Center for Global Health, Department of Medicine, Weill Cornell Medicine, New York, New York, United States of America; 8 National Institute of Science and Technology of Tropical Diseases (INCT-DT), Salvador, Bahia, Brazil; London School of Hygiene and Tropical Medicine, UNITED KINGDOM

## Abstract

**Background:**

Leprosy is a treatable infectious disease caused by *Mycobacterium leprae*. However, there is additional morbidity from leprosy-associated pathologic immune reactions, reversal reaction (RR) and erythema nodosum leprosum (ENL), which occur in 1 in 3 people with leprosy, even with effective treatment of *M*. *leprae*. There is currently no predictive marker in use to indicate which people with leprosy will develop these debilitating immune reactions. Our peripheral blood mononuclear cell (PBMC) transcriptome analysis revealed that activation of the classical complement pathway is common to both RR and ENL. Additionally, differential expression of immunoglobulin receptors and B cell receptors during RR and ENL support a role for the antibody-mediated immune response during both RR and ENL. In this study, we investigated B-cell immunophenotypes, total and *M*. *leprae*-specific antibodies, and complement levels in leprosy patients with and without RR or ENL. The objective was to determine the role of these immune mediators in pathogenesis and assess their potential as biomarkers of risk for immune reactions in people with leprosy.

**Methodology/findings:**

We followed newly diagnosed leprosy cases (n = 96) for two years for development of RR or ENL. They were compared with active RR (n = 35), active ENL (n = 29), and healthy household contacts (n = 14). People with leprosy who subsequently developed ENL had increased IgM, IgG1, and C3d-associated immune complexes with decreased complement 4 (C4) at leprosy diagnosis. People who developed RR also had decreased C4 at leprosy diagnosis. Additionally, elevated anti-*M*. *leprae* antibody levels were associated with subsequent RR or ENL.

**Conclusions:**

Differential co-receptor expression and immunoglobulin levels before and during immune reactions intimate a central role for humoral immunity in RR and ENL. Decreased C4 and elevated anti-*M*. *leprae* antibodies in people with new diagnosis of leprosy may be risk factors for subsequent development of leprosy immune reactions.

## Author summary

One in three people with leprosy develop an immune reaction, which worsen quality of life. Reactions occur despite effective treatment of the causative bacteria of leprosy, *Mycobacterium leprae*, with antibiotics. These reactions cause worsening skin lesions and additional nerve damage. Reactions can be chronic and recurrent, and may necessitate years of treatment with high dose corticosteroids. Our research focuses on why certain people develop these reactions while others do not. We found that people who have higher antibodies to *M*. *leprae* when they are diagnosed with leprosy are more likely to develop immune reactions in the two years after diagnosis. Also, we identify that C4 levels in the blood may be useful for monitoring the development and progression of leprosy reactions. These may be ways to identify who is at highest risk for leprosy immune reactions before they occur.

## Introduction

Leprosy, a chronic infectious disease caused by *Mycobacterium leprae*, is still a public health challenge for many countries, including Brazil [1]. There is a spectrum of leprosy that corresponds to the type of immune response elicited to *M*. *leprae*. Clinical, histopathologic, and mycobacteriologic criteria determine different clinical forms, formalized with the Ridley-Jopling criteria [2–5]. People with tuberculoid leprosy (TT) present with few hypopigmented, hypoesthetic skin lesions and little to no *M*. *leprae* on skin biopsy, whereas lepromatous patients (LL) have a stronger humoral immune response, more skin lesions and higher bacterial burden. Between these two polar forms, there are borderline forms of leprosy (BT, BB, and BL) [6]. The World Health Organization (WHO) developed a simpler classification to be applied in areas that lack the ability to complete histopathological studies and classifies leprosy as: paucibacillary (PB) if there are up to 5 lesions or a skin smear without acid-fast bacilli, and multibacillary (MB) if there are more than 5 lesions or acid-fast bacilli in a skin smear [1, 7, 8]. Generally, PB leprosy encompasses TT and BT clincial forms and MB includes BB, BL and LL clinical forms.

One-third of people with leprosy develop pathologic immune reactions, either reversal reaction (RR) or erythema nodosum leprosum (ENL) [9, 10]. RR is characterized by increased cell-mediated immune response [10, 11]. During ENL, there are tender subcutaneous nodules, systemic inflammation, and possible organ involvement [12, 13]. The intercurrence of leprosy reactions is directly associated with the morbidity of leprosy. ENL may be recurrent and chronic resulting in prolonged corticosteroids and/or thalidomide treatment, which brings significant additional side effects [11]. A recent study shows that people with ENL have significant reduction in quality of life scores related to physical function, bodily pain and general health when compared to leprosy patients without reaction [14].

In the transcriptome of peripheral blood mononuclear cells (PBMC), the classical complement canonical pathway is common to both RR and ENL [15]. Expression levels of immunoglobulin receptors and B cell receptors during RR and ENL support an antibody-mediated immune response during both RR and ENL [15]. One study demonstrated B cells in leprosy skin lesions [16]; however, the role of these cells in leprosy and reactions is still not entirely understood [16–18].

B-cells are activated by microorganisms via antigen-specific B-cell receptor (BCR) or non-specific pattern recognition receptors [19]. The activation threshold is decreased when complement receptor 2 (CD21) binds to immune complex (IC), stimulating antibody production [20]. Downregulation of B-cell activation occurs when antigen-bound IgG cross-links FcγRIIb (CD32B) and BCR [21]. Altered CD21 and CD32B have been linked to antibody-mediated autoimmune diseases, such as rheumatoid arthritis and systemic lupus erythematosus [22–24]. In this study, we assessed these markers of B-cell regulation, B cell immunophenotypes, and the levels of immunoglobulin subtypes and complement in leprosy patients with and without leprosy reactions with objective of determining their role in pathogenesis.

## Methods

### Study design

**Fig 1:**
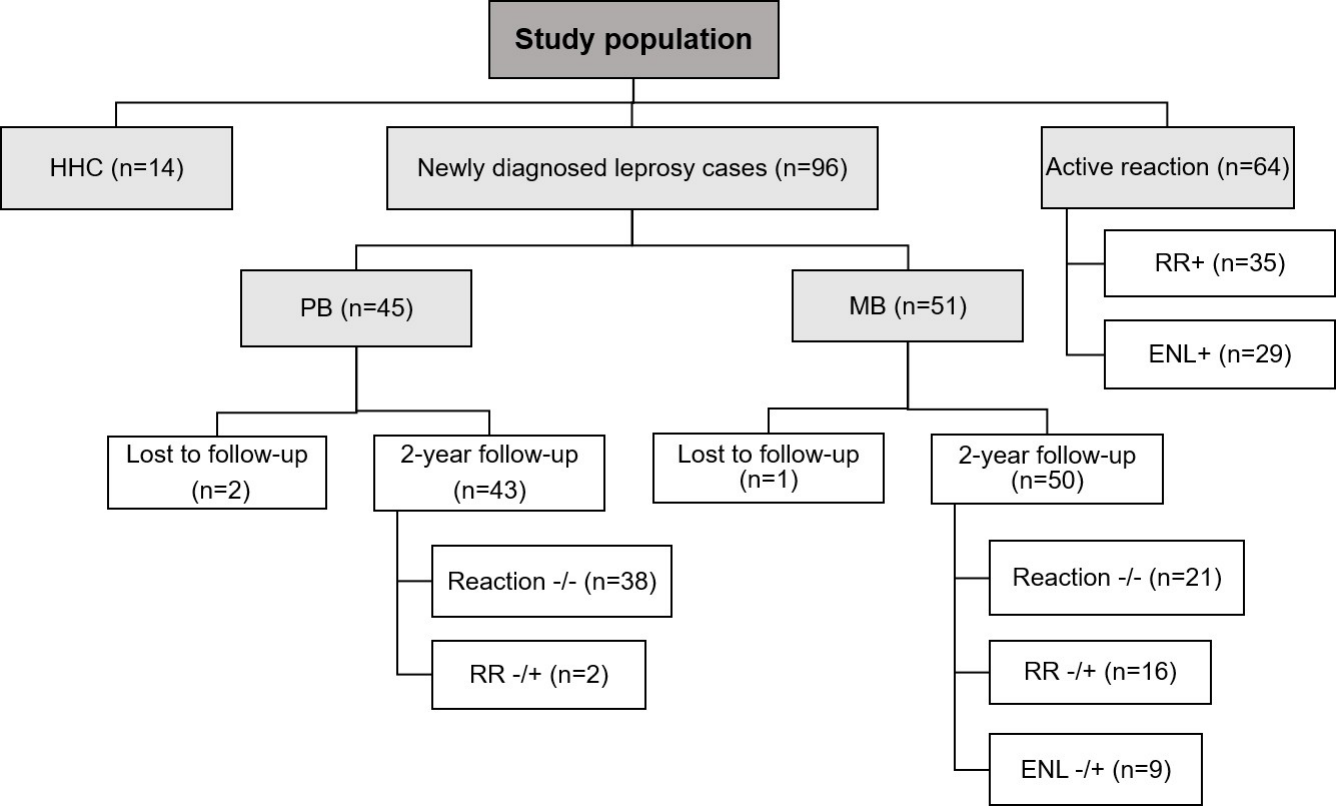
Flow chart of study enrollment and outcomes. HHC: household contacts; PB: paucibacillary leprosy; MB: multibacillary leprosy; RR+: active reversal reaction; ENL+: active erythema nodosum leprosum; Reaction -/-: patients that did not develop reactions during the follow-up; RR-/+: patient that developed RR during the follow-up; ENL-/+: patient that developed ENL during the follow-up. Isolated neuritis without cutaneous inflammation was not included as a “reaction” outcome.

Study participants 18 years of age or older were recruited in Natal, Brazil, from December 2010 to January 2017. Study enrollment is as shown in Fig 1. Individuals with pure neural leprosy, or who had received corticosteroid or thalidomide treatment in the 7 or 30 days, respectively, prior to blood collection were excluded from the study. Specimens at leprosy diagnosis were collected prior to initiation of multidrug therapy. Specimens at RR or ENL diagnosis were collected prior to initiation of immunomodulatory therapy

Diagnosis of leprosy was based on WHO criteria when one or more of the following cardinal signs were found: skin lesion consistent with leprosy with definite sensory loss, with or without thickened nerves and/or positive skin smears [25]. Classification of leprosy type by Ridley-Jopling criteria [6] was completed by a trained dermatologist (Mauricio L. Nobre or Maria do Carmo Palmeira Queiroz) using biopsy findings, bacterial index, and clinical findings [6]. As recommended by the Brazilian Ministry of Health, the bacterial index (BI) of each patient was calculated as a mean of the bacterial index obtained from four different areas of the body [26]. The standard regimen of multi-drug therapy (MDT) for leprosy in Brazil at the time of the study was dapsone daily and rifampicin monthly for PB leprosy. The regimen for MB leprosy was dapsone and clofazimine daily and rifampicin monthly, with dosages as recommended by the WHO. The non-leprosy control group included asymptomatic household contacts (HHC) of people with MB leprosy. All HHC underwent clinical examination and were advised to return to the hospital in case of any signs or symptoms of leprosy.

RR was clinically diagnosed when a leprosy patient presented with swelling, redness and warmth of skin patches, with or without tenderness of nerves and loss of their function. ENL was clinically diagnosed when the patient developed painful and red nodules under the skin, not associated with pre-existing leprosy lesions, usually accompanied by fever [27].

Newly diagnosed leprosy cases without RR or ENL were followed for 2 years, and then grouped by whether or not they developed RR or ENL during follow-up: 1.) No reaction (Reaction -/-); 2.) Developed RR (RR-/+); or **3**. Developed ENL (ENL-/+). The follow-up for reaction was performed monthly after leprosy diagnosis during MDT, and then was based on the review of each patient's medical record. Time to reaction was calculated as the time from leprosy diagnosis to development of the first reaction in the follow-up study period. RR or ENL may have had associated neuritis, but isolated neuritis without cutaneous inflammation was not included as a “reaction” outcome. For acute reactions, the groups were RR+ and ENL+.

### Ethical considerations

This study was reviewed and approved by the Ethics Committee of the Federal University of Rio Grande do Norte (approval 0042.0.051.051–09) and by the Institutional Review Board of Weill Cornell Medical College. All participants provided written informed consent.

### Peripheral blood mononuclear cell isolation and B cell typing by flow cytometry

Peripheral blood was collected in heparinized tubes and blood was diluted 1:1 with isotonic saline, applied to Ficoll-Paque Plus (GE Healthcare Life Sciences, Pittsburgh, PA), and centrifuged. The mononuclear cell layer was removed manually and washed with isotonic saline.

Antibody panels to define B cells were designed according to the Human Immunology Project recommendations [28]. Antibodies were obtained from BD Pharmigen (San Diego, CA, USA) unless specified otherwise. 250,000 PBMC were stained for 15 minutes with one of the following panels: **Panel 1**. anti-CD19-FITC (eBioscience, San Diego, CA, USA), anti-CD27-PE, anti-CD38-PE-Cy5, anti-CD24-PE-Cy7, anti-CD20-APC, and anti-CD3-APC-Cy7; and **Panel 2**. anti-CD32-FITC, anti-CD27-PE, anti-CD21-PECy5, anti-CD19-PE-Cy7, anti-CD20-APC, and anti-CD3-APC-Cy7 (S1 Table). For each panel, 50,000 events were acquired using a FACS-Canto II flow cytometer with FACSDiva software version 6.1.2 (Becton Dickson, Franklin Lakes, NJ, USA). Data were analyzed using FlowJo version 10.0.7 (TreeStar, Ashland, OR, USA). Lymphocyte populations were selected by forward-scatter and side-scatter profile, with B lymphocytes selected by CD19 positivity. Within the CD19^+^ B cells, we evaluated sub-populations as specified in the Human Immunology Project as well as the percentage of CD32^+^ and CD21^+^ cells [28]. The gating strategy is shown in S1 Fig.

### Quantification of immunoglobulins

Immunoglobulin types (total IgA, IgM, and IgE) and IgG subtypes (IgG2, IgG3, IgG4) were assayed with BD Cytometric Bead Arrays (San Diego, CA, USA). Data were acquired using a FACS-Canto II flow cytometer, recorded with FACSDiva software, and analyzed using FCAP Array software version 3.0 (BD, Franklin Lakes, NJ, USA). IgG1 was measured by enzyme-linked immunosorbent assay (ELISA) (Affymetrix eBioscience, Vienna, Austria) with IgG1 concentration determined by nonlinear regression of the standard curve using GraphPad Prism version 5.0 (San Diego, CA, USA).

### Detection of anti-*M*. *leprae* antibodies

Antibodies to the *M*. *leprae*-specific antigen “Leprosy IDRI Diagnostic-1 with Natural Disaccharide with Octyl ligation” (LID-NDO) were measured by ELISA, as previously described [29, 30]. Serologic results were assigned as positive or negative based on the laboratory’s receiver operating curve [29]. Raw optical density (OD) values were used as a continuous variable for regression analyses.

### Measurement of immune complexes and classical complement components

C3d-containing IC, C1q, C2, and C4 were quantified in serum by ELISA (Abcam, Cambridge, UK). Concentrations were calculated from standard curves using GraphPad Prism version 5.0.

### Immunohistochemistry of skin biopsies

Skin biopsies were collected into formalin and embedded in paraffin. Twenty micron sections were transferred to silanized glass slides (Starfrost, Knittel, Germany). Slides were deparaffinized and then subjected to antigen retrieval using a Dako (Carpinteria, CA) solution for 30 minutes at 95°C. Sections were incubated with rabbit monoclonal anti-human CD21 (clone EP3093; Abcam, Cambridge, MA), followed by secondary staining with goat anti-rabbit-AlexaFluor488 (Thermo Fisher Scientific, Rockford, USA). Nuclei were stained with 4’6-diamidino-2-phenylindole (DAPI, Sigma, St. Louis, MO) which selectively binds to the minor groove of double-stranded DNA [15]. Image capture, measurement of fluorescence, and C1q staining of these biopsies were as previously described [15]. Imaging and analyses were completed by investigators blinded to group assignment (Francianne M. Amorim and Marcos R. Costa).

### Statistical analysis

Analysis of Variance (ANOVA) or Kruskal-Wallis test followed by Tukey’s or Dunn's tests were used to compare differences between more than two groups (HHC, PB, and MB). To compare two groups (with or without reaction), double-sided Student's T-test and Mann-Whitney test were used for parametric or non-parametric data, respectively. Correlation analyses were performed using Spearman (bacterial index vs. frequency of B cells) or Pearson’s (bacterial index vs. anti-LID-1 ELISA OD) correlation tests.

Principal component analyses (PCA) were performed to assess the relationship of immunoglobulin levels overall in the 3 different phenotypes (HHC, PB, and MB). PCA was followed by MANOVA, and then one-way ANOVA if there was a difference between the profiles. The predictive value of LID-NDO ELISA optical density reading for reaction development was determined with binomial logistic regression models with response variable of (Reaction -/- vs. RR -/+) or (Reaction -/- vs. ENL) and with age and gender as covariates.

### Software

Analyses of noparametric data and graphics were performed with GraphPad Prism (version 5.0). Analyses of multivariate profiles and principal components were performed with Statistica (version 7.0). Binomial logistic regression models were generated with Stata (version 10.0). P-values ≤ 0.05 were considered statistically significant.

## Results

### Characteristics of the study population and outcomes after two-year follow up

Of the 96 newly diagnosed leprosy cases, 45 were classified as PB and 51 were MB (Fig 1; Table 1). Ninety-three of 96 people were followed for 2 years with 3 people lost to follow-up (n = 2 PB, n = 1 MB). An additional 64 leprosy patients with acute reaction (35 RR and 29 ENL) and 14 household contacts (HHC) of MB patients were enrolled. Because of limited sample availability, all participants were not included in all laboratory assays. The number of people in each group is included in the figure axis labels. The proportion of men was different in PB and MB groups (42.2% vs. 64.7%, respectively, p = 0.0274) (Table 1). While 51% of the new MB leprosy cases recruited without reaction had positive BI, 82.7% of the MB that were recruited during ENL had a positive BI at the leprosy diagnosis (Table 1). Of the 29 cases of ENL in MB, 25 had BI results and 4 did not have BI recorded. Of the 25 people with ENL and BI results, 24 of 25 had a positive BI. The person with an initial negative BI had the test repeated, which then yielded a positive BI. Of the MB patients, 73% had detectable anti-*M*. *leprae* antibodies, whereas 17.8% of the PB and 21.4% of HHC were seropositive.

**Table 1:** Characterization of the study population.

Variable	Controls	Newly diagnosed leprosy		MB leprosy with active reaction		
	HHC (n = 14)	PB (n = 45)	MB (n = 51)	RR (n = 35)	ENL (n = 29)	*p*-value
**Age, mean (± SD)**	42.9 (± 19.8)	44.9 (± 16.3)	52.1 (± 14.9)	51.8 (± 15.0)	45.6 (± 18.3)	0.0734^a^
**Male sex (%)**	5 (35.7)	19 (42.2)^b^	33 (64.7)^b^	21 (63.6)	19 (65.5)	0.0274^b^
**Ridley-Jopling**						
** TT**	-	34	0	0	0	-
** BT**	-	8	10	6	0	-
** BB**	-	0	14	16	0	-
** BL**	-	0	15	11	10	-
** LL**	-	0	8	0	16	-
** NA**	-	3	4	2	3	-
**Bacterial index**						
** Positive**	-	0	26	16	24	-
** Highly positive (BI ≥ 4)**	-	0	5	5	5	-
** NA**	-	7	2	2	4	-
**MDT status at recruitment**						
** Without**	-	45	51	14	3	-
** During**	-	0	0	15	9	-
**within first 6m**	-	-	-	12	4	-
** > 6 and < 12m**	-	-	-	3	5	-
** After**	-	0	0	6	17	-
**Anti-*M*. *leprae* positive (%)**	3 (21.4)	8 (17.8)	36 (70.6)	27 (77.1)	21 (72.4)	-

Abbreviations: HHC: household contact; PB: paucibacillary; MB: multibacillary; RR: reversal reaction; ENL: erythema nodosum leprosum; TT: tuberculoid; BT: borderline-tuberculoid; BB: borderline-borderline; BL: borderline-lepromatous; LL: lepromatous; NA: not available because histopathological analysis of lesions or slit skin smear were not performed; MDT: multidrug therapy. The bacterial index was calculated at leprosy diagnosis and represents the average of four different collection sites.

^a^ Analysis of variance (ANOVA)

^b^ Chi-square and Fisher's exact test.

Among PB leprosy who were followed for 2 years, 4.6% developed RR (2/43) (Fig 1). The development of RR in the PB group occurred in an average time of 141 days (minimum: 42; maximum: 241). In the MB group, 50% (25/50) had reactions: 32% (16/50) with RR and 18% (9/50) with ENL (Fig 1). RR developed an average of 199 (median: 157.5; minimum: 17; maximum: 503) days after leprosy diagnosis. ENL occurred at an average time of 322 (median: 319; minimum: 31; maximum: 714) days, respectively. In order to minimize variations inherent to leprosy clinical forms and considering that only two PB leprosy cases presented reaction during the follow-up, in the immunological studies of leprosy reactions we used only the newly diagnosed MB cases of the cohort (Reaction—/ -; RR—/ +; ENL—/ +). In addition, all cases with acute reaction (RR + or ENL +) recruited in our study were MB. As such, conclusions on RR and ENL are for people with MB leprosy and RR or ENL.

### B cell profile and expression of CD32 and CD21 in MB and PB leprosy

**Fig 2:**
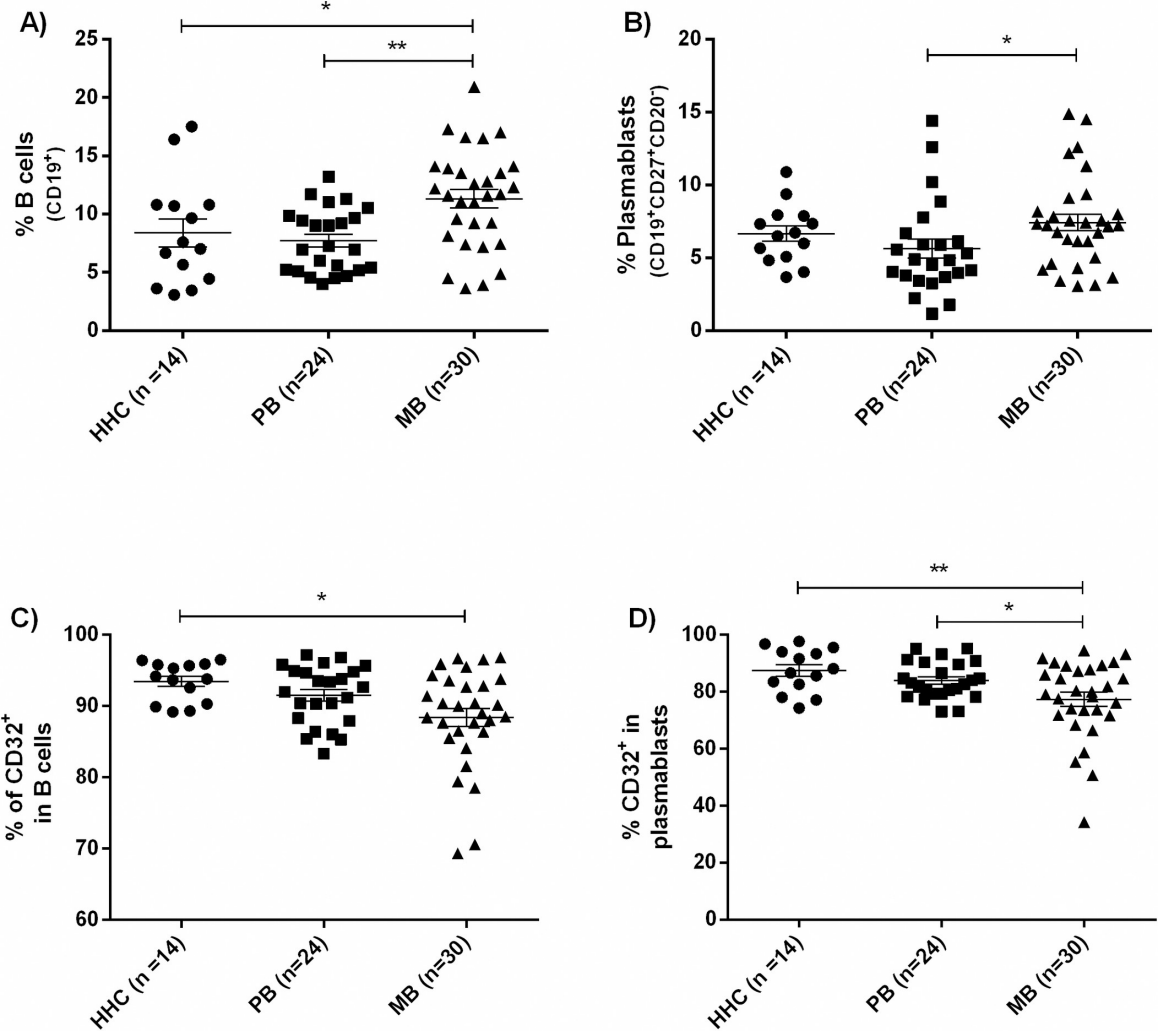
CD32 expression in B cells and plasmablasts. The frequency of CD19^+^ B cells as a subset of lymphocytes in the subtypes of leprosy are shown in **2A** with CD19^+^CD27^+^CD20^-^ plasmablasts as a subset of B cells in **2B**. Frequency of CD32B (FcγRIIb) in B cells (**2C**) and plasmablasts (**2D**) were decreased in MB leprosy compared to HHC. Comparison was with ANOVA followed by Tukey’s multiple comparisons test for **4A** and **4D**. Kruskal-Wallis followed by Dunn’s multiple comparisons test was used for **4B** and **4C**. * *p* ≤ 0.05, ** *p*< 0.01. The horizontal bar is the mean and the vertical bars the standard error of the mean (SEM).

MB leprosy had a higher frequency of CD19^+^ B cells in the mononuclear cell population than HHC or PB leprosy (11.3% vs. 8.4% vs. 7.7%, p = 0.003, Fig 2A). There was a correlation between CD19^+^ B cell abundance and bacterial index (Spearman’s correlation: r = 0.2487, p = 0.045). MB patients had a greater frequency of plasmablasts (CD19^+^CD27^+^CD20^-^) when compared to PB (7.4% vs. 5.6%, p = 0.0318) (Fig 2B). There was no difference in the frequency of transitional B cells (CD19^+^CD24^high^CD38^high^) or memory B cells (CD19^+^CD27^+^CD20^+^) between groups (S2 Fig). There was also a decreased frequency of CD32^+^ plasmablasts in MB patients when compared to HHC and PB (77.4% vs. 87.5% vs. 83.9%, p = 0.0082; Fig 2D). There were no differences in the frequency of CD21^+^ B cells, memory B cells or plasmablasts or CD32^+^ memory B cells (S3 Fig).

### Immunoglobulin and complement levels in MB and PB leprosy

IgG1 was increased in MB patients when compared to PB (p = 0.0428), S2 Table, and correlated with the bacterial index in MB leprosy (r = 0.4245, p = 0.0031). IgG2 was reduced in both PB and MB leprosy compared to HHC (p = 0.0176). For the other immunoglobulins, no differences were observed between PB and MB leprosy and HHC. In a principal component analyses, considering all immunoglobulins, principal component (PC) 1 and PC2 explained 42% of the total data variation by leprosy subtype. MANOVA showed a difference in the profile of PC between the phenotypes (HHC, PB and MB) (Wiks λ = 0.8480; p = 0.0381). MB patients had increased levels of IC and C2 when compared to PB leprosy (S2 Table). There was no difference in C1q or C4 levels between the groups.

### Profile of B cells and expression of CD32 and CD21 during leprosy reactions

**Fig 3:**
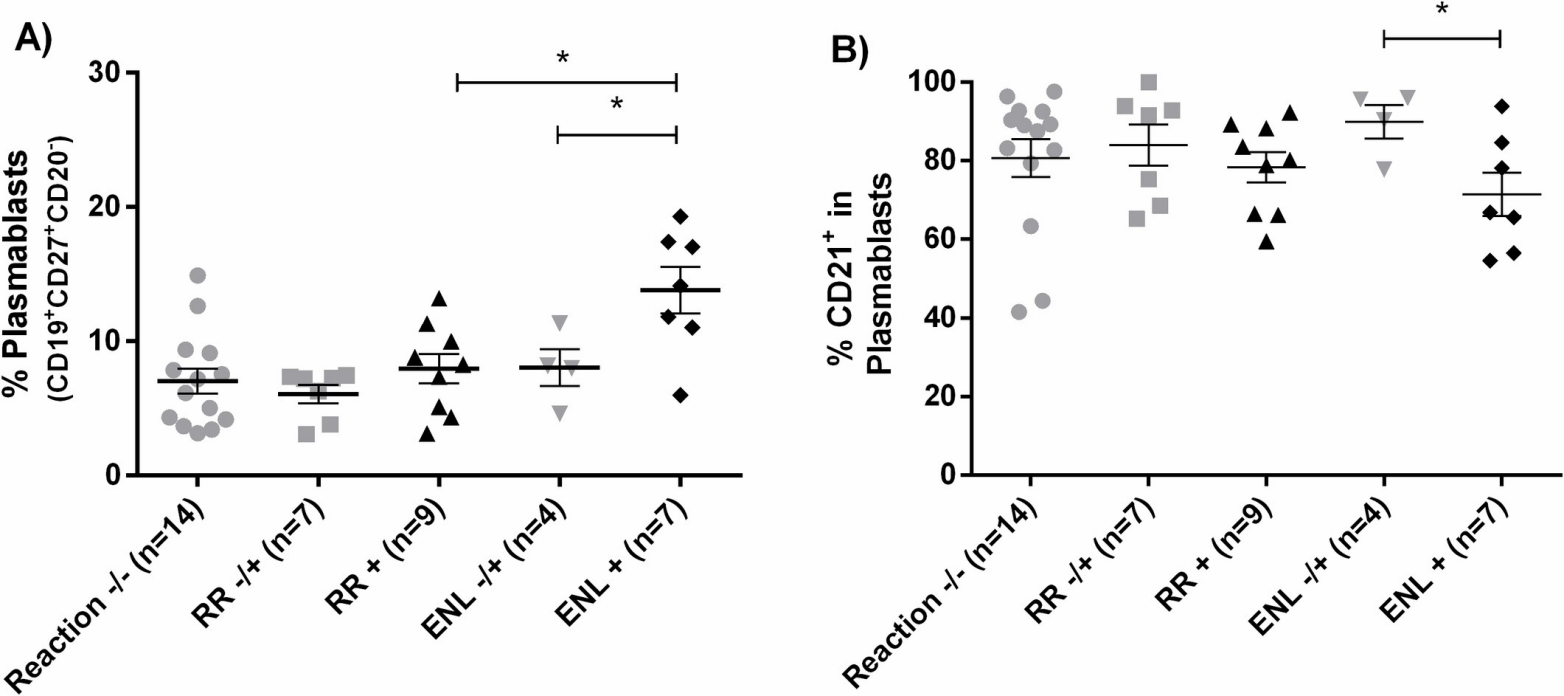
Plasmablast populations and CD21 expression in leprosy immune reactions. Plasmablast populations were proportionally greater in people with ENL (**3A**) and fewer of these plasmablasts were CD21+ compared to non-reaction groups (**3B**). Comparison was made between MB patients that do not develop immune reactions in 2-years of follow-up (Reaction-/-) with those with MB who developed RR (RR-/+) or ENL (ENL -/+) (ANOVA or Kruskal-Wallis test). Those patients who developed reactions during the cohort were also compared with patients with active immune reactions (RR+ and ENL+) (Student’s t test or Mann-Whitney test). * *p* ≤ 0.05. The horizontal bar is the mean and the vertical bars the standard error of the mean (SEM).

Immunologic studies of RR and ENL outcomes were limited to people with 2 years of follow-up and multibacillary leprosy only, given the differences between PB and MB leprosy and the small number of RR and lack of ENL in people with PB leprosy. There was no difference in B cell phenotypes between MB patients who developed or did not develop RR or ENL. However, during active ENL (ENL+) there was an increased frequency of plasmablasts (13.8%) in the blood when compared to patients at leprosy diagnosis who went on to develop ENL later (ENL-/+) (8%, p = 0.0497) (Fig 3A). There was no statistically significant difference in the frequency of CD32^+^ B cells, but ENL+ presented decreased frequency of CD21^+^ B cells when compared to ENL-/+ (80.4% vs. 90.6%, p = 0.0129), including in plasmablasts (71.5% vs 89.9%, p = 0.0481; Fig 3B).

**Fig 4:**
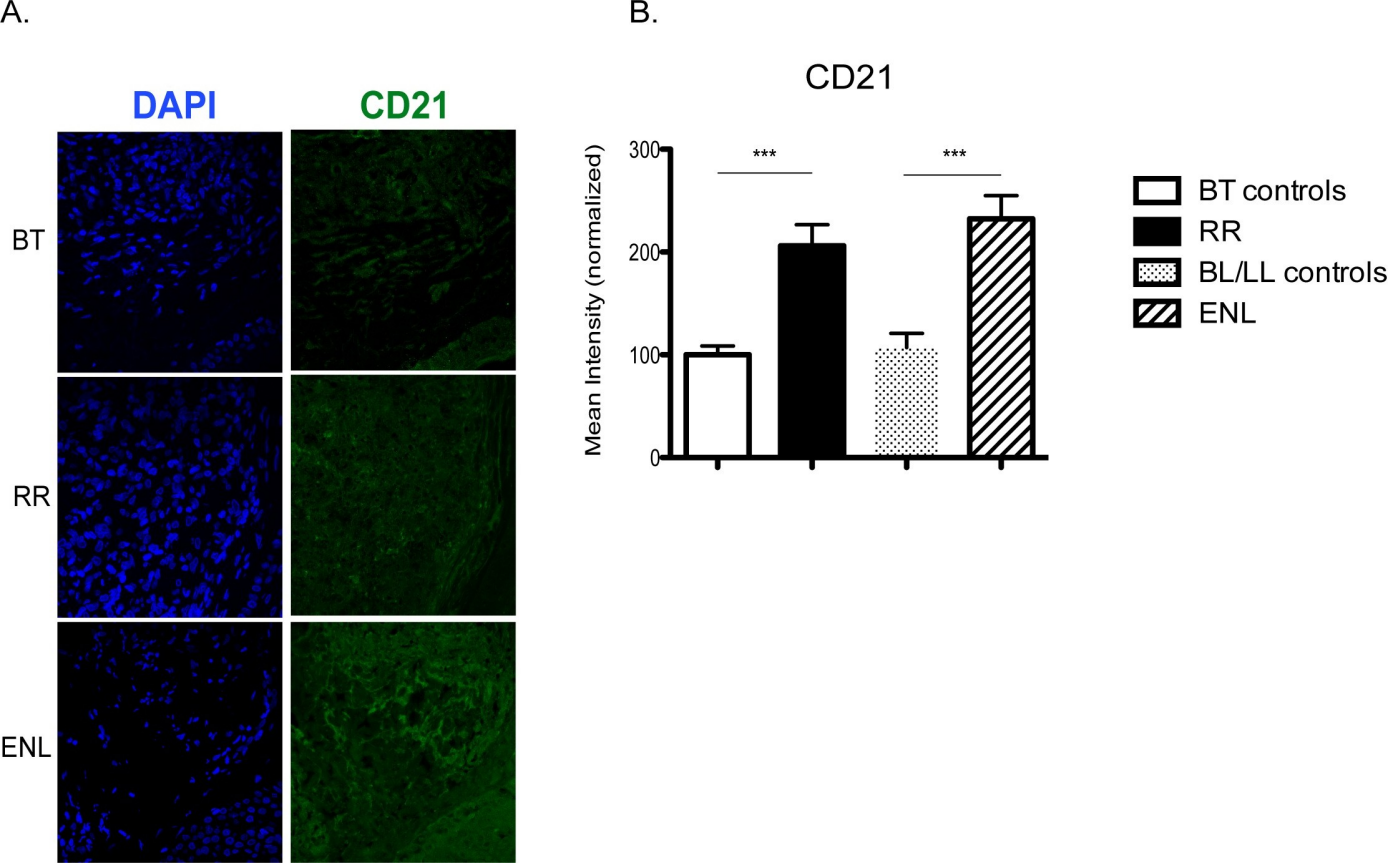
CD21 in the skin lesions of leprosy patients. The figure shows representative photos (**4A**) and quantitative measurement of fluorescence (**4B**). Each photo is a compilation of 15 slices of 1.25 μM confocal micrographs, as previously described [15]. Nuclei were stained with 4’6-diamidino-2-phenylindole (DAPI). Individuals with active RR (n = 3) and active ENL (n = 3) were compared with individuals with the same clinical forms of leprosy but without reactions: BT controls (n = 7) and BL/LL controls (n = 3). Differences in fluorescence between groups were assessed with ANOVA followed by Tukey multiple-comparison test. *** *p*< 0.001. The horizontal bars represent means and the vertical bars the standard error of the mean (SEM).

To evaluate whether this decrease in CD21^+^ B cells in the blood of ENL+ could be related to an increase in these cells in the skin lesion tissue, we measured the expression of CD21 in skin lesions of a set of leprosy patients with active RR (n = 3), active ENL (n = 3), and no reaction (BT, n = 7; BL/LL, n = 3). We found that CD21 levels in the skin were statistically significantly higher in patients with active ENL and active RR when compared to patients without reactions (p<0.0001; Fig 4A and 4B), suggesting that more CD21^+^ cells are present in skin lesions during immune reactions.

### Immunoglobulins, immune complexes and complement proteins during leprosy reactions

**Fig 5:**
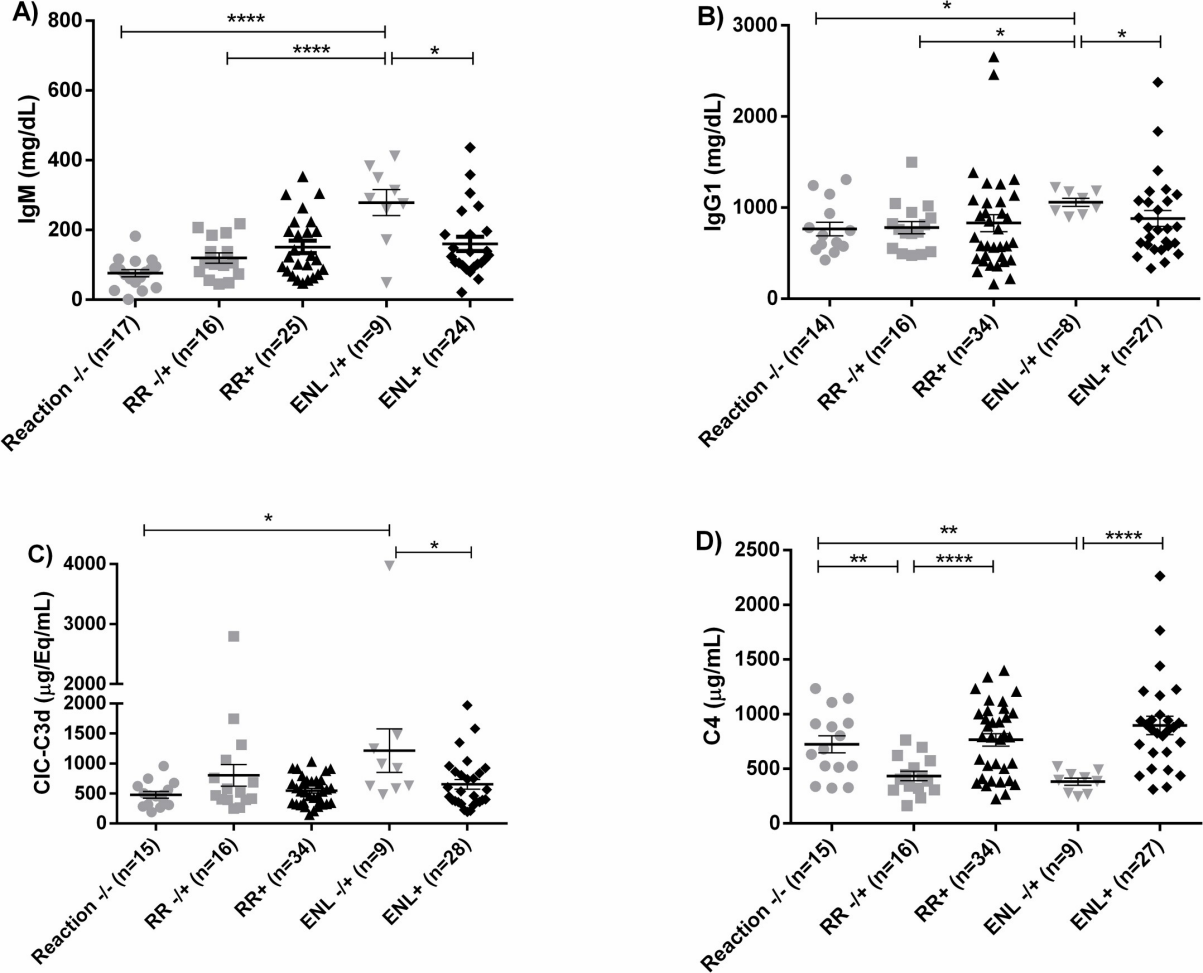
Immunoglobulins and complement components in leprosy and leprosy reactions. IgM levels were higher in ENL -/+ at the time of leprosy diagnosis, but the mean IgM level was lower during acute ENL (**5A**) with a similar pattern for IgG1 levels (**5B**). C3d-CIC were increased in people at leprosy diagnosis who went on to develop ENL (**5C**). C4 was lower in RR -/+ and ENL -/+, but higher during acute RR and acute ENL (**5D**). Comparison was made between MB patients that do not develop immune reactions in a 2-years follow-up (Reaction-/-) with those that developed RR (RR -/+) or ENL (ENL -/+) during the follow-up (ANOVA or Kruskal-Wallis test). Those patients that developed reactions during the follow-up were also compared with patients with active presentation of immune reactions (RR+ and ENL+) (Student’s t test or Mann-Whitney test). *, *p* ≤ 0.05, **, *p* < 0.01, ****, *p* < 0.0001. The horizontal bars represent mean value and the vertical bars the standard error of the mean (SEM).

At leprosy diagnosis, people who went on to develop ENL (ENL -/+) had increased levels of IgM when compared to RR or no reaction (278.3 vs. 151.3 vs. 76.15 mg/dL, respectively; p<0.0001) (Fig 5A). A similar pattern was seen for IgG1, with higher levels in ENL -/+ (1059mg/dL) than in RR -/+ (780.6mg/dL), or Reaction -/- (766.2mg/dL, p = 0.0183) (Fig 5B). ENL -/+ also had increased levels of C3d-associated IC at leprosy diagnosis when compared to Reaction -/- (1213 vs. 478.5μg/Eq/mL, p = 0.0140) (Fig 5C). Interestingly, there was decrease in total IgM (p = 0.0122), IgG1 (p = 0.0457) and C3d-associated IC (p = 0.0250) during acute ENL when compared to ENL -/+ (Fig 5A and 5B).

During active RR, there was increased serum C4 compared to pre-RR (RR+ vs. RR -/+, p<0.0001; Fig 5D). In active ENL, there was also increased C4 compared to pre-ENL (ENL+ vs. ENL -/+, p = 0.0018; Fig 5D). Both RR -/+ and ENL -/+ groups had lower C4 at leprosy diagnosis when compared to MB patients who did not develop reactions (p = 0.0004; Fig 5D).

### Predictive value of anti-LID-NDO serology for reaction risk in MB patients

**Fig 6:**
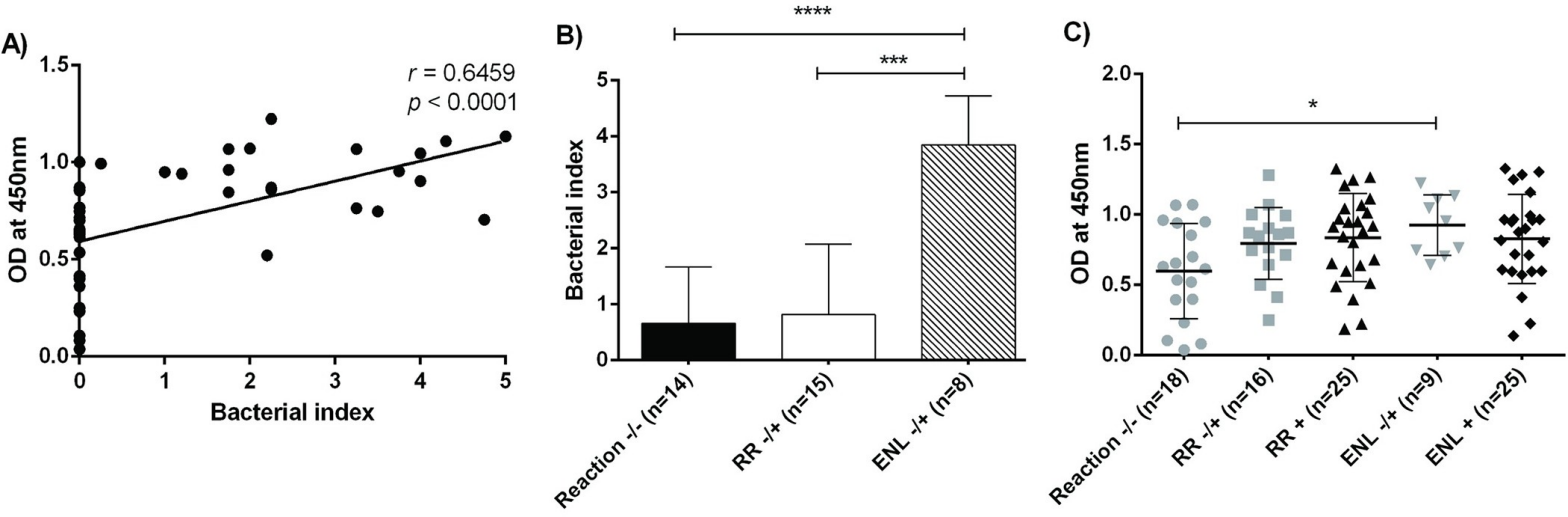
Anti-*M*. *leprae* antibodies in the peripheral blood of MB patients prior to and and during leprosy reactions. The OD of LID-NDO ELISA was positively correlated with *M*. *leprae* bacterial index by dermal smears (Pearson’s correlation, p<0.0001) (**6A**). The bacterial index at diagnosis was higher in those patients who developed ENL during the follow-up (ENL-/+) when compared to the bacterial index of people who did not develop reactions (Reaction -/-) or those who developed RR (RR-/+) (Kruskal-Wallis test followed by Dunn multiple comparison test, p<0.001) (**6B**), as was the LID-NDO OD (ANOVA followed by Dunn multiple comparison test, p≤0.05) (**6C**). In Fig **6C**, the horizontal bars represent mean value and the vertical bars the standard error of the mean (SEM). * *p* ≤ 0.05, *** *p*< 0.001, **** *p*< 0.0001.

The anti-LID-NDO OD were positively correlated with the bacterial index (BI) of MB patients (r = 0.6459; p<0.0001) (Fig 6A). On average, ENL -/+ had a higher BI than RR -/+ or Reaction -/- (BI = 3.8 vs. 0.8 vs. 0.67, respectively; p<0.001; Fig 6B). ENL -/+ had higher anti-LID-NDO antibody levels at leprosy diagnosis when compared to Reaction -/- (mean OD = 0.9246 vs. 0.5975, p = 0.0220) (Fig 6C). However, there was no difference between RR-/+ and Reaction -/- (mean OD = 0.7955 vs. 0.5975, p = 0.0657).

The predictive value of LID-NDO serology for development of reactions was assessed by logistic binomial models. In the first model, patients with higher level of LID-NDO OD had a greater risk of RR (OR = 11.27; p = 0.020; 95% CI [1.47–86.27]); that is, for each 0.1 increase in the LID-NDO OD, the risk of RR increased by 12.7%. Likewise, in the second model, patients with higher LID-NDO OD had a greater risk of ENL (OR = 12.77; p = 0.021; 95% CI [1.47–110.64]); that is, for each 0.1 increase in the LID-NDO OD, the risk of ENL increased by 27.7%. Sex and age were not statistically significantly associated with development of RR or ENL during 2 years of follow-up.

## Discussion

The objective of this study was to determine the biological correlates of gene expression differences in immunoglobulin- and complement-related canonical pathways during leprosy immune reactions [15] by characterizing B cell phenotypes, and quantitate general and anti-*M*. *leprae-*specific immunoglobulins and complement proteins in the blood.

We found decreased frequency of CD32B in plasmablasts of MB patients; this decrease in negative feedback could contribute to increased antibody production. This corresponds to the increased LID-NDO OD we observed in people with MB leprosy. We observed that 21.4% of our HHC had positive serology for *M*. *leprae*, indicating a high environmental exposure to the *M*. *leprae*, a percentage similar to that observed in a previous study [29]. However, this positivity does not invalidate their use as controls since we sought to compare individuals with leprosy and non-diseased individuals, and all HHC were clinically evaluated and were considered healthy and free of signs of symptoms and signs of leprosy. In B cells of healthy people, CD32B binding down-regulates antibody production and decreases antibody-independent functions of B cells [31]; the decrease in CD32B we saw could contribute to increased antibody production, as well as non-antibody immune effects. Additionally, downregulation of CD32 on B cells has been reported in autoimmune diseases [22, 23]. We hypothesize that a dysregulation of CD32 may contribute to persistence of *M*. *leprae* infection as the humoral response is augmented but insufficient to control bacterial growth, and to the intensified immune response which manifests as RR and ENL.

In our cohort, we observed that only 4.6% of PB cases had a reaction during follow-up, which is likely related to the large number of TT cases, who only rarely develop RR. Among MB leprosy cases, the incidence of reactions was much higher, similar to that observed by Hungria *et al* in their cohort involving 753 cases of leprosy [32].

People with MB leprosy had increased IgG1, with higher levels in pre-ENL MB leprosy. Class switching to IgG1 is usually triggered by protein antigens [33], which may be related to the positive correlation between *M*. *leprae* bacterial index and IgG1 level in MB leprosy in this study. Among the IgG subclass, IgG1 is a major inducer of CD32b-mediated negative regulation [34], which may reflect counter-regulation of B cells.

We saw elevated anti-LID-NDO antibodies, as determined by OD, in people at leprosy diagnosis who went on to develop RR or ENL in the next 2 years. The predictive value of anti-PGL-1 levels for leprosy immune reactions is unclear [32, 35–37]. However, we saw a high and statistically significant risk of reaction development with increase in anti-LID-NDO at leprosy diagnosis. Hungria *et al* also reported a significant difference at baseline anti-LID-NDO antibody levels between patients that do not developed reactions and those that developed RR or ENL during follow-up (34). However, the accuracy of the LID-NDO serology, calculated by ROC curve, was considered satisfactory only for predictive analysis of ENL (AUC = 0.799) (34). Serrano-Coll *et al* observed an increase in the seropositivity rate and in the magnitude of anti-LID-NDO response among leprosy patients with history of reactions (without differentiating RR and ENL) when compared to those without history of reaction at the end of MDT [38]. In our cohort, there was a correlation between bacillary load on dermal smear and anti-LID-NDO optical density on ELISA. Identifying specific targets of the antibodies circulating in the blood at increased levels in leprosy and leprosy reactions will help to determine if the antigenic stimulus in RR and ENL is host, *M*. *leprae*, or another pathogen. A potential future direction is antibody sequencing of people with RR and ENL.

We hypothesized that immunoglobulins could have a role in the pathogenesis of both RR and ENL, based on pathways analysis of PBMC transcriptome data [15]. Here, we observed that MB patients who subsequently developed ENL already had increased levels of IgM, IgG1, and C3d-associated IC at leprosy diagnosis, compared to MB patients who did not develop reaction. These results suggest that risk factors for subsequent ENL may be present at disease diagnosis, and thus could be used to estimate risk of developing reactions.

The percentage of circulating B cells that are plasmablasts is increased during ENL, but there is a decreased frequency of circulating CD21^+^ B cells. B cells can be part of the inflammatory infiltrate in skin lesions of people with BT, BL and LL leprosy [16], but the role of these cells in the pathogenesis of leprosy and ENL needs to be better understood. A possible explanation for decreased CD21^+^ B cells in peripheral blood during ENL is that there could be migration of CD21^+^ B cells to the tissues where they secrete antibodies. This hypothesis was supported by increased CD21 staining in skin during ENL. We also found decreased IgG1 and IC levels in the blood during acute ENL, which could be related to IC formation and deposition in tissues. This is supported by a study reporting lower blood IgG1 levels during acute ENL [39].

Interestingly, people with MB leprosy who subsequently developed reactions had lower levels of C4 at leprosy diagnosis than people who did not develop reactions. In those who subsequently developed reactions, C4 increased during RR or ENL compared with pre-reaction levels. Our previous study showed increased expression of C1q in PBMC and increased deposition of C1q in skin lesions, during both RR and ENL [15]. Here, we did not observe increased levels of C1q in the blood; this could be due to deposition in tissues during reactions. Deposition of C1q can occur with IgG1, IgG2 and IgG3, but IgG1 and IgG3 are the most effective [40, 41]. In our analysis, IgG1 was increased in sera of MB patients, but decreased during ENL, which supports a C1q-IgG1 deposition hypothesis. Further studies are indicated to determine the components of immune complexes in skin lesions of ENL and RR.

In summary, our data suggest that the humoral immune response of MB patients is associated with numerical and functional changes in B cells, which may have a role in pathophysiology of reactions. A weakness of this study is that we do not have extensive information about immune cells and immune complexes in the skin during the immune reactions. Additionally, we were unable to compare humoral immune response of the same individuals prior to and at the time of RR or ENL. One limitation of this study was the small number of paired pre vs. post-reaction samples, related to the short interval between reaction diagnosis and initiation of immunomodulatory medications that precluded inclusion in the study. However, we clearly observed that patients with elevated levels of anti-LID-NDO antibodies at diagnosis of leprosy were at high risk of subsequently developing leprosy reactions. In addition, we demonstrated decreased level of C4 in the blood preceding development of reactions and increased levels during their acute presentation, suggesting that this protein could be considered as a diagnostic marker for leprosy immune reaction (RR and ENL). The additional evidence for immunoglobulin and complement involvement in RR and ENL presented here, in addition to recent findings [18, 42], support the hypothesis of a central role for humoral dysregulation in development of the pathologic immune reactions of leprosy.

Since leprosy reactions represent the main determinant of leprosy associated morbidity, the identification of risk groups for the development of leprosy reactions by clinicians, while allowing a more intensive follow-up, is of fundamental importance in reducing their incidence, as well as reducing the establishment of permanent physical disabilities. Other cohort studies involving a larger population may contribute to the validation of the predictive value of the serological tests tested here. Serological tests such as those performed here do not require a large workforce, whereas their cost can be justified if the results of them can be used to reduce the costs of prolonged treatment to corticosteroid and thalidomide, in addition to improving quality of life of the population affected.

## Supporting information

S1 Checklist STROBE checklist. (XLSX)

S1 Fig Gating strategy. Lymphocytes were selected in a forward and side scatter diagram and B cells were selected by CD19 staining. Within the B cell population its sub-populations were analysed as transitional B cells (CD19^+^CD24^high^CD38^high^), naïve B cells (CD19^+^CD27^-^CD20^+^), memory B cells (CD19^+^CD27^+^CD20^+^) and plasmablasts (CD19^+^CD27^+^CD20^-^).
(TIF)

S2 Fig **Frequency of transitional (A) and memory (B) B cells in the peripheral blood of paucibacillary (PB) and multibacillary (MB) leprosy and household contacts (HHC).** For comparison between the three groups Kruskal-Wallis followed by Dunn’s multiple comparisons test was used. The horizontal bars represent mean value and the vertical bars the standard error of the mean (SEM).
(TIF)

S3 Fig Frequency of CD21 (CR2) in B cells, memory B cells and plasmablasts in paucibacillary (PB) and multibacillary (MB) leprosy and household contacts (HHC). For comparison between the three groups Kruskal-Wallis followed by Dunn’s multiple comparisons test was used. The horizontal bars represent mean value and the vertical bars the standard error of the mean (SEM).
(TIF)

S1 Table Antibodies used in flow cytometry. (DOCX)

S2 Table Concentrations of immunoglobulin classes and subclasses, immune complexes (IC) and complement proteins. Abbreviations: HHC, household contact; PB, paucibacillary; MB, multibacillary; MB with RR, multibacillary with reversal reaction; MB with ENL, multibacillary with erythema nodosum leprosum. The concentrations are represented as mean ± standard error of mean. The superscript letters are indicating between which two groups are the statistical differences observed.
(DOCX)
