# Supplementary figures and images for: Differential immunoglobulin and complement levels in leprosy prior to development of reversal reaction and erythema nodosum leprosum
Source: PLoS Negl Trop Dis. 2019 Jan 28;13(1):e0007089. doi: 10.1371/journal.pntd.0007089 (PMC6366718; doi:10.1371/journal.pntd.0007089)

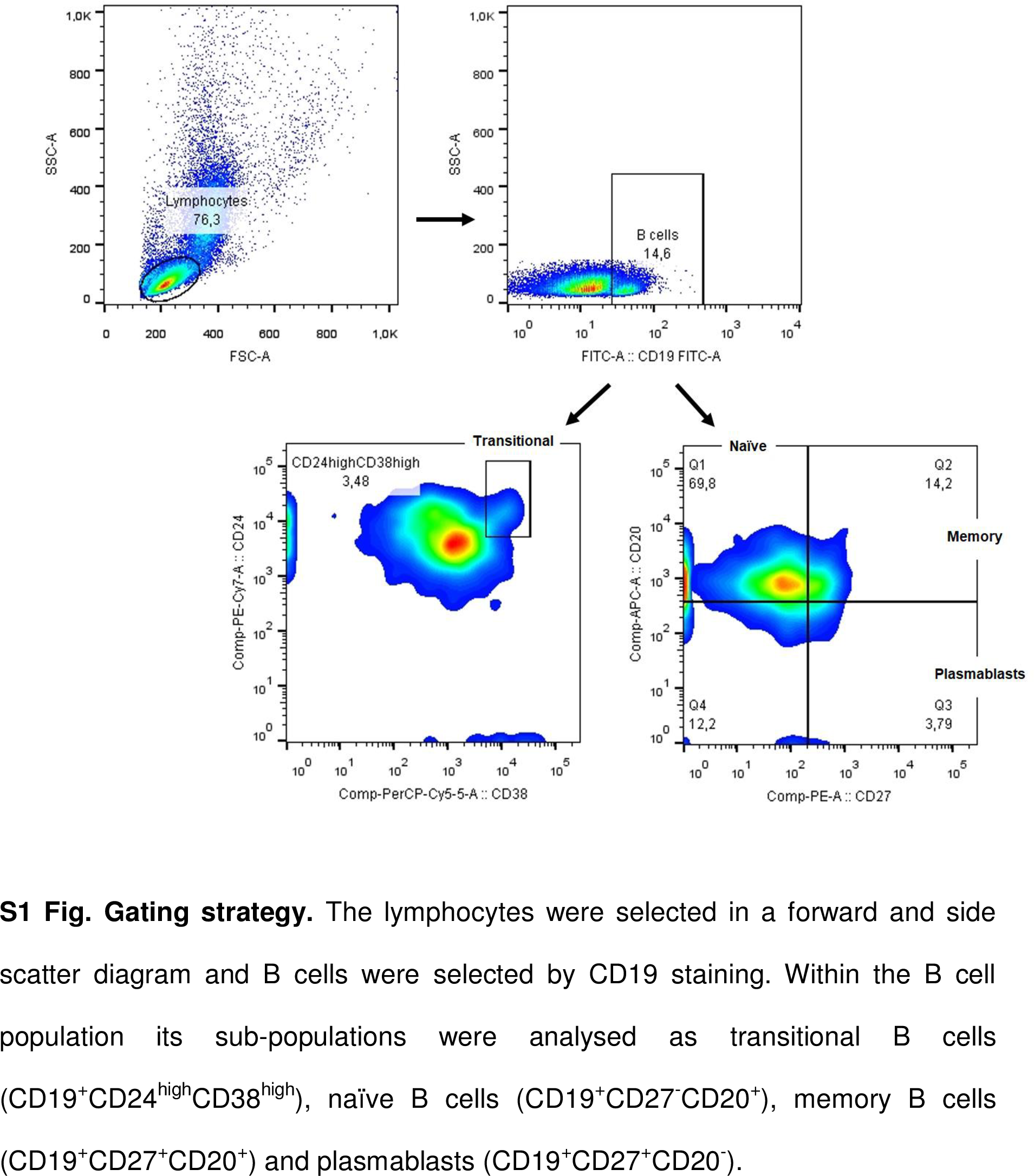

Supplement: S1 Fig — Lymphocytes were selected in a forward and side scatter diagram and B cells were selected by CD19 staining. Within the B cell population its sub-populations were analysed as transitional B cells (CD19+CD24highCD38high), naïve B cells (CD19+CD27-CD20+), memory B cells (CD19+CD27+CD20+) and plasmablasts (CD19+CD27+CD20-). (TIF) [file pntd.0007089.s002.tif]

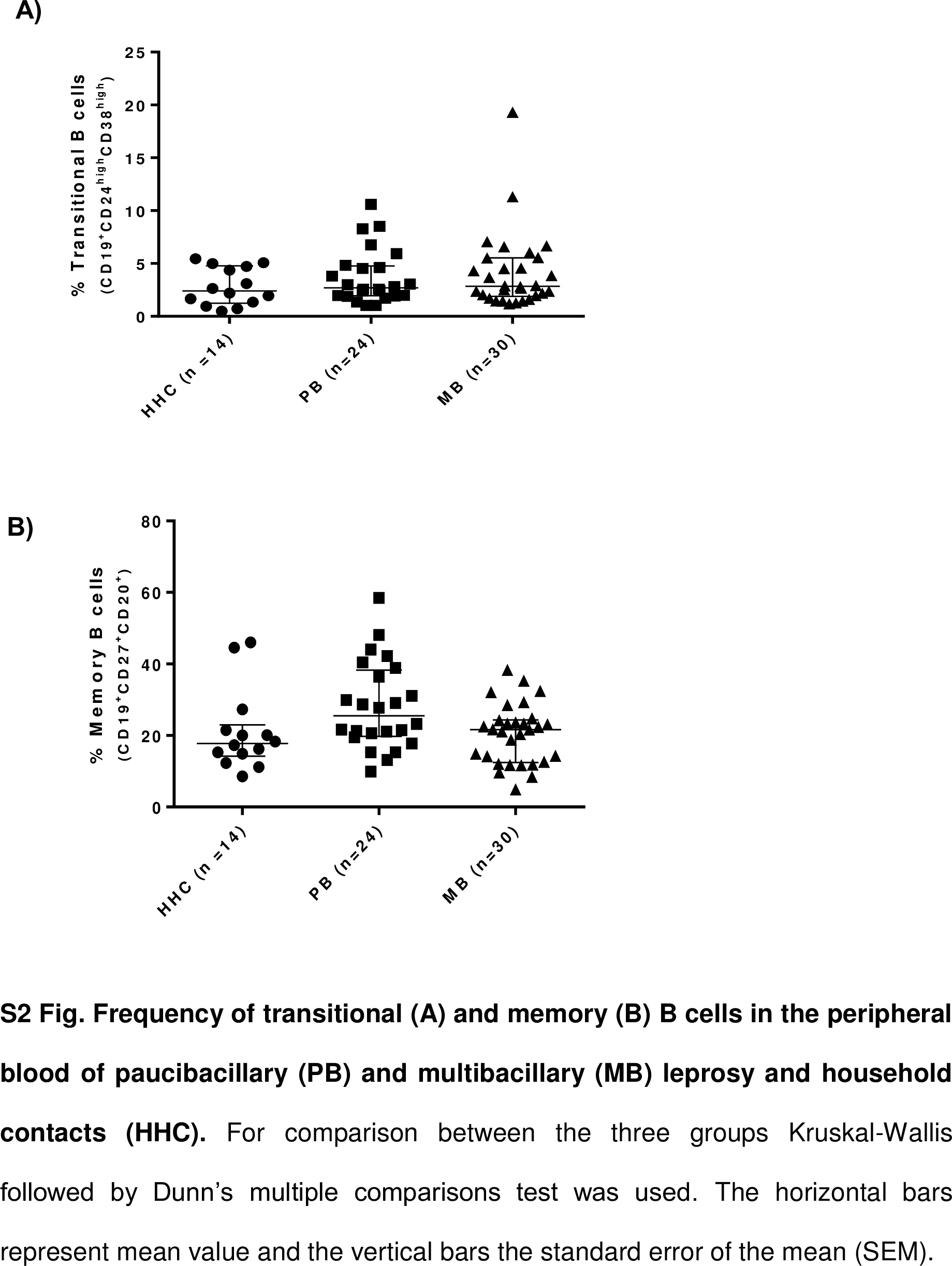

Supplement: S2 Fig — Frequency of transitional (A) and memory (B) B cells in the peripheral blood of paucibacillary (PB) and multibacillary (MB) leprosy and household contacts (HHC). For comparison between the three groups Kruskal-Wallis followed by Dunn’s multiple comparisons test was used. The horizontal bars represent mean value and the vertical bars the standard error of the mean (SEM). (TIF) [file pntd.0007089.s003.tif]

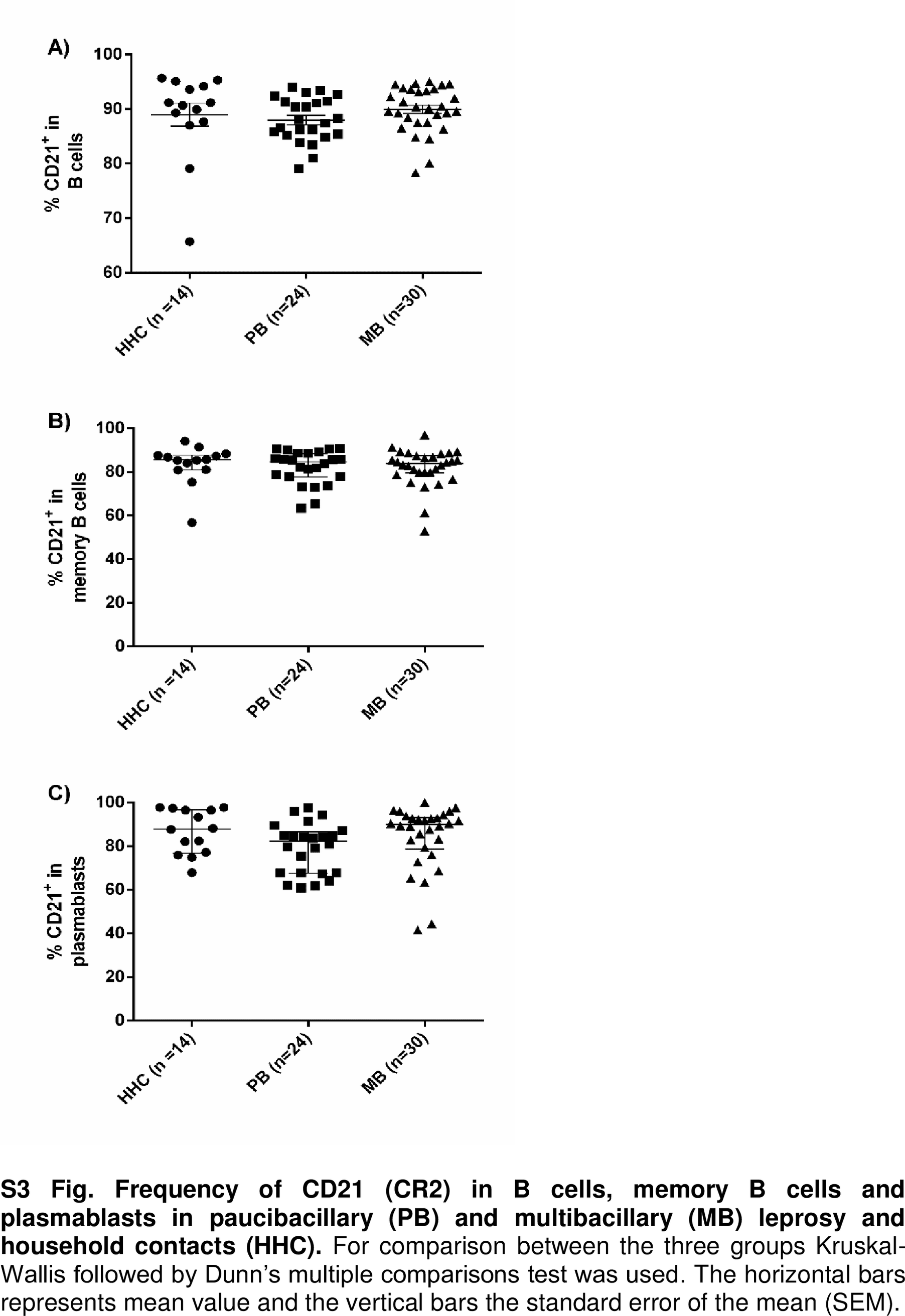

Supplement: S3 Fig — For comparison between the three groups Kruskal-Wallis followed by Dunn’s multiple comparisons test was used. The horizontal bars represent mean value and the vertical bars the standard error of the mean (SEM). (TIF) [file pntd.0007089.s004.tif]
