# Supplementary material for: Differential immunoglobulin and complement levels in leprosy prior to development of reversal reaction and erythema nodosum leprosum
Source: PLoS Negl Trop Dis. 2019 Jan 28;13(1):e0007089. doi: 10.1371/journal.pntd.0007089 (PMC6366718; doi:10.1371/journal.pntd.0007089)
Supplement: S1 Table — (DOCX) [file pntd.0007089.s005.docx]

**S1 Table. Antibodies used in flow cytometry.**

| Antibody | Clone | Panel |
| --- | --- | --- |
| Anti-CD19-FITC | HIB19 | 1 |
| Anti-CD27-PE | M-T271 | 1 and 2 |
| Anti-CD38-PE-Cy5 | HIT2 | 1 |
| Anti-CD24-PE-Cy7 | ML5 | 1 |
| Anti-CD20-APC | 2H7 | 1 and 2 |
| Anti-CD3-APC-Cy7 | SK7 | 1 and 2 |
| Anti-CD32-FITC | 3D3 | 2 |
| Anti-CD21-PE-Cy7 | B-ly4 | 2 |
| Anti-CD19-PE-Cy7 | SJ25C1 | 2 |
