# Supplementary material for: Differential immunoglobulin and complement levels in leprosy prior to development of reversal reaction and erythema nodosum leprosum
Source: PLoS Negl Trop Dis. 2019 Jan 28;13(1):e0007089. doi: 10.1371/journal.pntd.0007089 (PMC6366718; doi:10.1371/journal.pntd.0007089)
Supplement: S2 Table — Abbreviations: HHC, household contact; PB, paucibacillary; MB, multibacillary; MB with RR, multibacillary with reversal reaction; MB with ENL, multibacillary with erythema nodosum leprosum. The concentrations are represented as mean ± standard error of mean. The superscript letters are indicating between which two groups are the statistical differences observed. (DOCX) [file pntd.0007089.s006.docx]

**S2 Table.** **Concentration of immunoglobulin classes and subclasses, immune complexes (IC) and proteins of the complement**

| Immunoglobulin | | HHC | PB | MB | p-value |
| --- | --- | --- | --- | --- | --- |
| IgM (mg/dL) | 168.7 ± 22.4 | | 113.7 ± 10.4 | 136.1 ± 15.5 | 0.0733 |
| IgG1 (mg/dL) | 727.7 ± 90.5 | | 697.3 ± 47.8^a^ | 830.1 ± 43^a^ | *0.0428* |
| IgG2 (mg/dL) | 690.2 ± 168.5^a,b^ | | 426.2 ± 61.2^a^ | 413.7 ± 73^b^ | *0.0176* |
| IgG3 (mg/dL) | 134.8 ± 40.7 | | 128.7± 27.2 | 169.6 46.4 | 0.9501 |
| IgG4 (mg/dL) | 96.1± 55.5 | | 75.02 ± 19.7 | 81 ± 19.9 | 0.8854 |
| IgA (mg/dL) | 433.3 ± 102.7 | | 346.8 ± 27 | 378.6 ± 27.9 | 0.6604 |
| IgE (ng/mL) | 117.4 ± 30 | | 230 ± 54.9 | 194.9 ± 24.1 | 0.2266 |
| IC (μg/Eq/mL) | 512 ± 111.4 ^a^ | | 434 ± 33.5 ^b^ | 748.6 ± 105.7 ^a,b^ | *0.0107* |
| C1q (μg/mL) | 373 ± 49 | | 350.6 ± 30.8 | 307.5 ± 24.7 | 0.4389 |
| C2 (μg/mL) | 46.9 ± 3.2 | | 40.3 ± 1.5^a^ | 48.1 ± 2.3^a^ | *0.0248* |
| C4 (μg/mL) | 471.8 ± 60.1 | | 511.6 ± 37.9 | 520.1 ± 39.1 | 0.8516 |

Abbreviations: HHC, household contact; PB, paucibacillary; MB, multibacillary; MB with RR, multibacillary with reversal reaction; MB with ENL, multibacillary with erythema nodosum leprosum. The concentrations are represented as mean ± standard error of mean. The superscript letters are representing statistical difference between two groups in each analysis.
